# Clinical value of the nomogram model based on endoscopic ultrasonography radiomics and clinical indicators in identifying benign and malignant lesions of the pancreas

**DOI:** 10.3389/fonc.2025.1504593

**Published:** 2025-02-19

**Authors:** Xiaofei Fan, Jia Huang, Xiaohan Cai, Ayixie Maihemuti, Shu Li, Weili Fang, Bangmao Wang, Wentian Liu

**Affiliations:** Department of Gastroenterology, Tianjin Medical University General Hospital, Tianjin, China

**Keywords:** pancreatic lesions, endoscopic ultrasonography, radiomics, clinical indicators, nomogram

## Abstract

**Objective:**

Based on endoscopic ultrasonography (EUS) radiomics and clinical data, we constructed a radiomics model and a nomogram model for identifying benign and malignant pancreatic lesions, and explored the diagnostic performance of these two prediction models.

**Methods:**

Images and clinical data of 151 patients with pancreatic lesions detected by EUS from January 2018 to September 2023 were retrospectively collected. The patients were randomly divided into a training set and a validation set at a ratio of 7:3. Through feature extraction and feature screening of EUS images, we calculated the radiomics score (rad-score) to realize the construction of the radiomics model. Collecting the clinical data, laboratory test results, and rad-scores from patients, univariate and multivariate logistic regression analyses were used to screen statistically significant influencing factors that could help identify benign and malignant lesions of the pancreas, and a nomogram model was constructed. The diagnostic performance and clinical utility of the two prediction models were evaluated using the receiver operating characteristic (ROC) curves, calibration curves, and decision curve analysis (DCA).

**Results:**

Through feature extraction and screening, eight non-zero coefficient features were finally selected to calculate the rad-score. Multivariate logistic regression analysis showed that rad-score, age, and CA199 were the influencing factors in predicting benign and malignant pancreatic lesions. A nomogram model was constructed based on the three factors. In the validation set, the nomogram model exhibited superior performance with an AUC = 0.865 (95% CI 0.761–0.968) compared to the radiomics prediction model. The calibration curve and DCA depicted that the nomogram model demonstrated superior accuracy and yielded a higher net benefit for clinical decision-making compared to the radiomics prediction model.

**Conclusion:**

Based on EUS radiomics and clinical indicators, we constructed a promising nomogram model to accurately identify benign and malignant pancreatic lesions.

## 1 Introduction

Pancreatic cancer (PC), benign pancreatic tumor (BPT), and mass-forming pancreatitis (MFP) are common pancreatic lesions (1–4). They often present with clinical manifestations such as jaundice, weight loss, and abdominal pain (5). However, their treatment methods and prognoses differ significantly. To prevent overtreatment, early and accurate differentiation between benign and malignant pancreatic lesions is critical. At present, endoscopic ultrasonography (EUS) has become one of the most important examination methods for diagnosing pancreatic lesions. It can not only allow for the visualization of lesion size and depth, assessment of surrounding lymph nodes and vascular invasion, and evaluation of adjacent tissue and organ involvement, but also provide the possibility for cellular and histological diagnosis through endoscopic ultrasound-guided fine-needle aspiration (EUS-FNA) and endoscopic ultrasound-guided fine-needle biopsy (EUS-FNB) (6). However, some studies indicate that the specificity of EUS for diagnosing pancreatic malignancy is limited, with figures as low as 58% (7). Even after the injection of contrast, the manifestations of different pancreatic lesions are also non-specific and there may be overlapping appearance (8). Meanwhile, the diagnostic accuracy of EUS is closely related to the endoscopist’s knowledge, experience, and operational level, with poor inter-observer agreement (9). Relying solely on EUS images to distinguish between benign and malignant lesions can be subjective and challenging for endoscopists (10). Pathologic biopsy is the gold standard for diagnosis. The diagnostic rate of EUS-FNA for pancreatic lesions is approximately 70%–82%, and the diagnostic accuracy of EUS-FNB is approximately 70%–89% (11–13). However, they are invasive procedures that may result in complications such as infection, bleeding, abdominal pain, and self-limited pancreatitis (14). Therefore, a safe, effective, simple, and objective way to identify benign or malignant pancreatic lesions is needed.

The concept of radiomics, first proposed by Lambin et al. in 2012, is an emerging computer-aided diagnosis (CAD) technology that has been widely used in recent years for tumor research (15–17). Based on radiomics and ultrasound images, it has been widely used in the diagnosis of thyroid tumors, breast tumors, etc. (18–21). However, there are relatively few studies investigating the application of EUS radiomics for distinguishing between benign and malignant pancreatic lesions; thus, this study constructed a radiomics prediction model and a nomogram prediction model based on EUS radiomics and clinical data to distinguish benign and malignant pancreatic lesions.

## 2 Materials and methods

### 2.1 Participant identification

**Figure 1:**
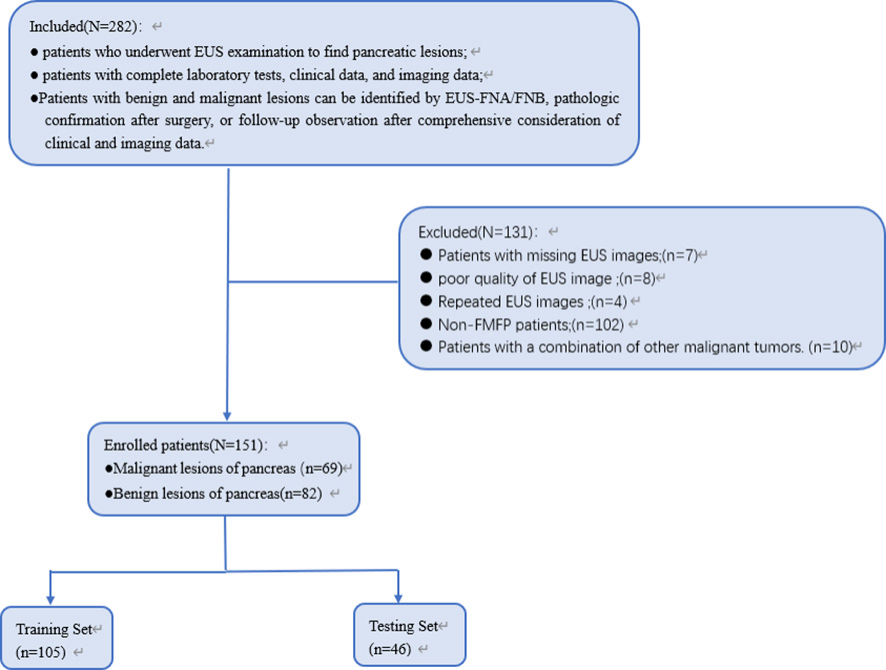
Flowchart of patient selection.

This study retrospectively included clinical data and images from 151 patients with pancreatic lesions detected by EUS at the Gastrointestinal Endoscopy Center of the General Hospital of Tianjin Medical University from January 2018 to September 2023. Among these patients, 69 cases were classified as malignant and 82 were classified as benign. The patients were randomly divided into a training set (*N* = 105) and a validation set (*N* = 46) at a 7:3 ratio. The inclusion criteria were as follows (1): patients who underwent EUS examination to find pancreatic lesions (2); patients with complete laboratory tests, clinical data, and imaging data; and (3) patients with benign and malignant lesions that can be identified by EUS-FNA/FNB, pathologic confirmation after surgery, or follow-up observation after comprehensive consideration of clinical and imaging data. The exclusion criteria were as follows (1): patients with missing EUS images (2); EUS images quality is poor and cannot be used for analysis (3); repeat EUS examinations for the same patient, selecting only the clearest image with the largest measured section (4); non-focal MFP (non-FMFP) patients; and (5) patients with a combination of other malignant tumors. In this study, patient data were anonymized, and all patients’ personal information was deleted from the final results. This study is a retrospective study and has been approved by the Tianjin Medical University General Hospital Medical Research Ethics Committee and Institutional Review Board, exempting informed consent. The patient selection flowchart is shown in **Figure 1**.

### 2.2 Construction of the radiomics model

#### 2.2.1 EUS image acquisition

Patients were examined using radial EUS (GF-UCT240-AL5; Olympus Medical, Tokyo, Japan) and fan-scan EUS (GF-UE260-AL5; Olympus Medical, Tokyo, Japan). Contraindications to EUS and puncture were excluded preoperatively. Patients and their families were fully informed of the risks associated with the operation and signed informed consent forms. An experienced EUS physician performed a continuous scan of the pancreas to explore the lesion site, measure the lesion length and diameter, and observe the image characteristics of the lesion and its relationship with the surrounding organs and blood vessels. If there was any disagreement, it was confirmed by a senior EUS physician. The images were acquired in accordance with the current quality control indexes and standardized specifications. The EUS images were saved to the Picture Archive and Communication System (PACS) and the clearest images containing the largest section of the pancreatic lesion were selected and stored in BMP (Bitmap) format.

#### 2.2.2 Workflow

**Figure 2:**
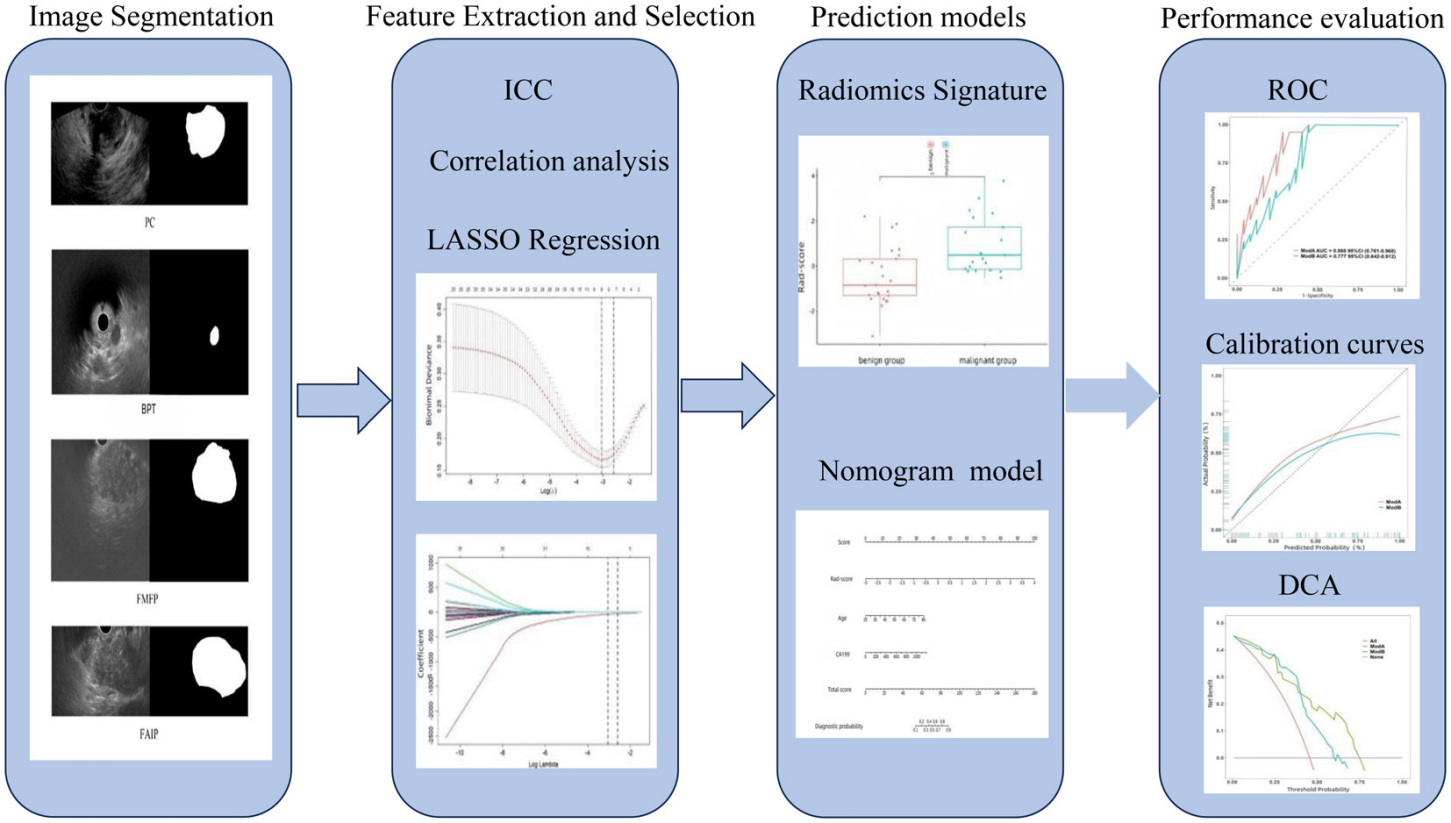
Flowchart of the radiomics analysis.

The workflow for the radiomics analysis consisted of lesion segmentation, feature extraction, feature selection, model construction, and evaluation (**Figure 2**).

#### 2.2.3 Lesion segmentation and preprocessing

The acquired EUS images were imported into COCO Annotator software, and the regions of interest (ROIs) were manually outlined along the tumor boundaries by an experienced EUS physician and confirmed by a senior EUS physician. The above images were processed in grayscale.

#### 2.2.4 Feature extraction and feature selection

Image radiomics features were automatically extracted using Python 3.9.0 and Pyradiomics v3.0.1. In this study, 1,032 radiomics features were extracted from the ROI of each EUS image, which can be divided into eight types according to image types: Original, Exponential, Gradient, LocalBinaryPattern2D, Logarithm, Square, Squareroot, and Wavelet (this includes LH, HL, HH, and LL). The above radiomics features can be further divided into three categories based on feature types (1): shape features (2), first-order histogram features, and (3) second-order histogram features, including gray-level co-occurrence matrix features, gray-level dependency matrix features, gray-level run length matrix features, gray-level size zone matrix features, and neighborhood gray-level zone difference matrix features.

Intraclass and interclass correlation coefficients (ICCs) were used to assess intra-observer and inter-observer reliability. A set of 30 EUS images was randomly chosen. An experienced EUS physician (A) and a senior EUS physician (B) independently performed ROI segmentation. Two weeks later, physician A repeatedly performed the ROI segmentation. Radiomics features with the ICC value greater than 0.70 (indicating excellent stability) were selected for subsequent analysis.

Pearson correlation analysis and the least absolute shrinkage and selection operator (LASSO) logistic regression algorithm were applied for feature selection. Firstly, Pearson correlation coefficients were calculated to assess feature correlation. When the correlation coefficient between any two features exceeded 0.7, it indicated a strong correlation, which could lead to multicollinearity problems in the model. To enhance the accuracy of data analysis, these highly correlated and redundant features were removed. Finally, the 10-fold cross-validation was used to identify radiomics features with non-zero coefficients using the LASSO regression model.

#### 2.2.5 Radiomics model construction

Finally, the features with predictive value were selected, and the radiomics score (rad-score) was calculated through coefficient weighting to construct the radiomics model.

### 2.3 Construction of the nomogram model

Medical records were reviewed to gather each patient’s clinical information, laboratory test results, and characterization of pancreatic lesions based on EUS examination. The variables included (1) gender (2); age (3); CA199 (4); lesion size, defined as the diameter of the largest section of the lesion (5); cystic–solid appearance of lesions: “solid” indicates that the lesion has a purely parenchymal component and shows echogenic manifestations such as very hypoechoic, moderately hypoechoic, and hypoechoic echoes on EUS; “cystic” indicates that the lesion has a purely fluid component and shows echo-less areas on EUS; “mixed” refers to lesions with both fluid and parenchymal components, displaying both echo-less and echogenic areas on EUS; and (6) lesion location: lesions in the head and neck are categorized as the pancreatic head and neck group; lesions in the body and tail are categorized as the pancreatic body and tail group; if the lesions are large or distributed across multiple sites, they are categorized as the total pancreas group. Clinical indicators and rad-scores were selected through univariable and multivariable logistic regression in the training set to construct the nomogram prediction model.

### 2.4 Statistical analysis

Data conforming to normal and approximately normal distributions are expressed as mean and standard deviation. Non-normally distributed continuous data were expressed as the median (upper and lower quartiles). Categorical data were expressed as frequencies and percentages. We compared variables of the participants utilizing an independent-sample *t*-test, Mann–Whitney *U* test, or χ^2^ test, where appropriate. The study was statistically analyzed using the R software package (version 4.2.1). Receiver operating characteristic (ROC) curves were plotted to evaluate the diagnostic efficacy of the models, and area under the curve (AUC) comparisons were performed using the DeLong test. Plotting calibration curves to evaluate the calibration of the model and decision curve analysis (DCA) was used to assess the clinical efficacy of the predictive models. A *p*-value of less than 0.05 was considered statistically significant.

## 3 Results

### 3.1 Clinical characteristics

In this study, 151 patients with pancreatic lesions were finally included, including 69 PC and 82 benign lesions. Clinical data, laboratory test results, and characteristics of pancreatic lesions of each patient were obtained by reviewing the medical records. Gender was not statistically different between the two groups of patients (*p* = 0.484). Age, CA199, lesion size, cystic–solid appearance of the lesion, and lesion site were statistically different between the two groups of patients (*p* < 0.05). Details are shown in **Table 1**.

**Table 1:** Clinical characteristics in all patients.

Characteristics	All (*N* = 151)	Benign (*N* = 82)	Malignancy (*N* = 69)	*p*-value
Gender (%)				0.484
Male	73 (48)	37 (45)	36 (52)	
Female	78 (52)	45 (55)	33 (48)	
Age (years), mean ± standard deviation	59.78 ± 11.69	56.93 ± 12.62	63.17 ± 9.49	<0.001*
CA199 (U/mL), median (upper and lower quartiles)	98.23 (7.84, 318.41)	15.01 (4.58, 318.41)	318.41 (82.63, 1,200)	<0.001*
Lesion size (cm), mean ± standard deviation	3.08 ± 1.80	2.44 ± 2	3.85 ± 1.14	<0.001*
Cystic–solid appearance of the lesion (%)				<0.001*
Solid	94 (62)	37 (45)	57 (83)	
Cystic	42 (28)	39 (48)	3 (4)	
Mixed	15 (10)	6 (7)	9 (13)	
Lesion site (%)				0.007*
Head and neck	78 (52)	43 (52)	35 (51)	
Body and tail	54 (36)	23 (28)	31 (45)	
Total pancreas	19 (13)	16 (20)	3 (4)	

*Significant difference *p*-value < 0.05.

Comparing the clinical indexes of patients in the benign and malignant groups in the training set, the results showed that the difference in gender between the two groups was not statistically significant (*p* = 0.594), but the differences in age, CA199, lesion size, cystic–solid appearance of the lesion, and lesion site between the two groups were statistically significant (*p* < 0.05), as shown in **Table 2**.

**Table 2:** Clinical characteristics in the training set.

Characteristics	All (*N* = 105)	Benign (*N* = 57)	Malignancy (*N* = 48)	*p*-value
Gender (%)				0.594
Male	55 (52)	28 (49)	27 (56)	
Female	50 (48)	29 (51)	21 (44)	
Age (years), mean ± standard deviation	59.92 ± 12.08	57.07 ± 12.87	63.31 ± 10.21	0.007*
CA199 (U/mL), median (upper and lower quartiles)	167.71 (13.67, 318.41)	21.58 (4.53, 318.41)	318.41 (167.22, 1,200)	<0.001*
Lesion size (cm), mean ± standard deviation	3 ± 1.80	2.32 ± 1.98	3.81 ± 1.11	<0.001*
Cystic–solid appearance of the lesion (%)				<0.001*
Solid	69 (66)	27 (47)	42 (88)	
Cystic	28 (27)	26 (46)	2 (4)	
Mixed	8 (8)	4 (7)	4 (8)	
Lesion site (%)				0.025*
Head and neck	54 (51)	29 (51)	25 (52)	
Body and tail	37 (35)	16 (28)	21 (44)	
Total pancreas	14 (13)	12 (21)	2 (4)	

*Significant difference *p*-value < 0.05.

### 3.2 Radiomics model results

**Figure 3:**
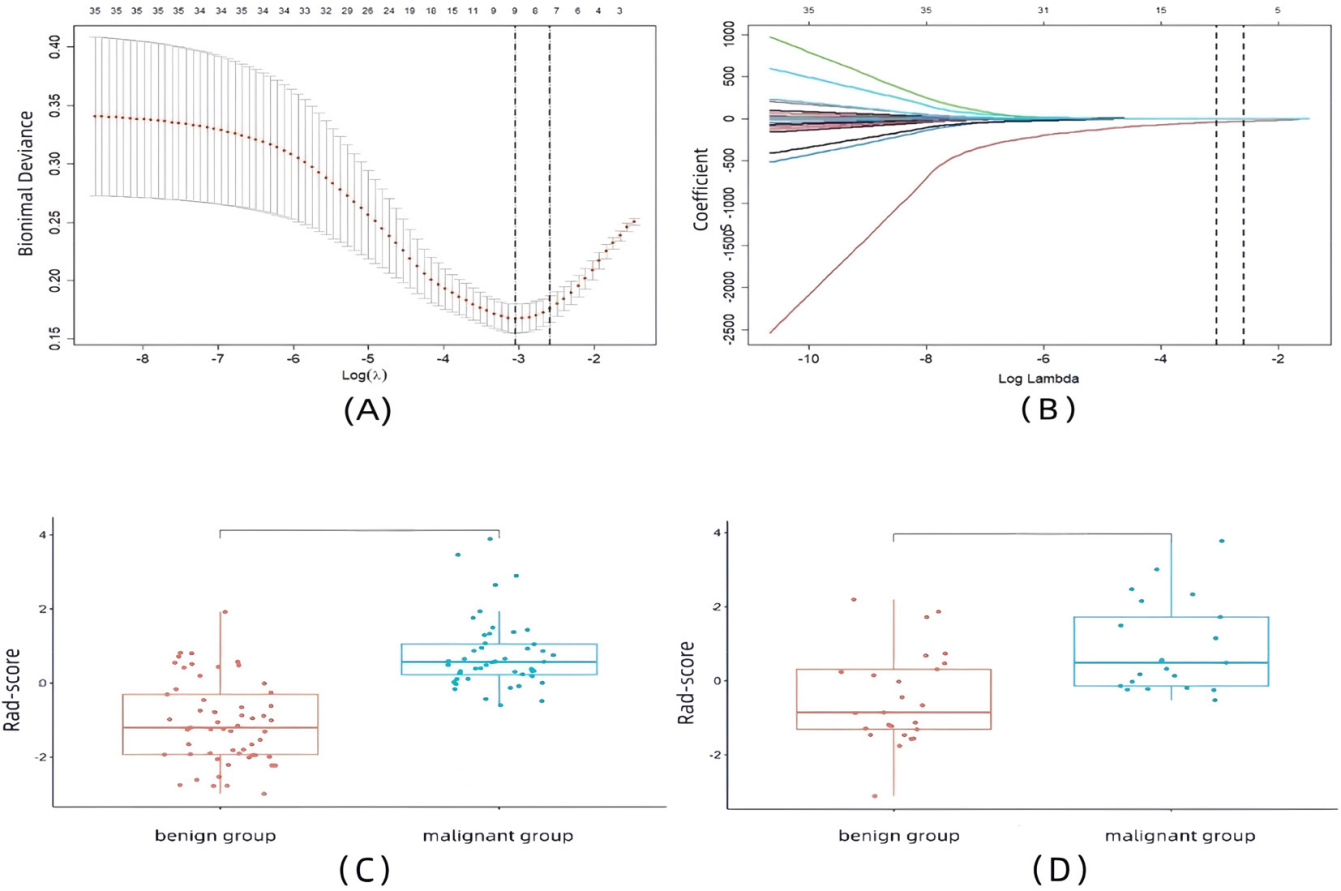
Radiomics feature selection and rad-scores results. **(A)** Selection of the tuning parameter (λ) in the LASSO model via 10-fold cross-validation based on minimum criteria. **(B)** LASSO coefficient profile plot with different log (λ) was displayed. **(C)** Box plots of rad-scores in the training set. **(D)** Box plots of rad-scores in the validation set.

The ROI of each EUS image in this study was extracted to 1,032 features, of which 582 features had an ICC greater than 0.8. After eliminating features with a correlation coefficient greater than 0.7, 35 features remained. We performed LASSO regression analysis with 10-fold cross-validation on the retained features, and finally selected eight non-zero coefficient features with the highest predictive value, including one morphological feature, one first-order histogram feature, and six second-order histogram features, as detailed in **Figures 3A, B**. A linear combination of these eight features and their corresponding weighted coefficients was used to generate the rad-score calculation formula, resulting in a rad-score for each patient to construct the final radiomics predictive model. The linear expression of the radiomics model is:

Rad-score = (−2.493685e−01) + (4.457582e−01) × (original_shape2D_Elongation) + (1.824497e−02) × (original_glszm_GrayLevelNonUniformity) + (9.148610e-01) × (gradient_glcm_Imc1) + (9.552690e−07) × (gradient_glszm_LargeAreaHighGrayLevelEmphasis) − (1.068615e−01) × (gradient_glszm_SizeZoneNonUniformity) − (4.128836e-01) x (lbp.2D_firstorder_10Percentile) + (4.416447e−02) × (square_glszm_GrayLevelNonUniformity) − (4.088332e+01) ×(squareroot_ngtdm_Contrast)

The results showed that the rad-scores of the malignant group were higher than those of the benign group in both the training set and the validation set (*p* < 0.001, *p* = 0.001), as shown in **Figures 3C, D**.

### 3.3 Nomogram model results

**Figure 4:**
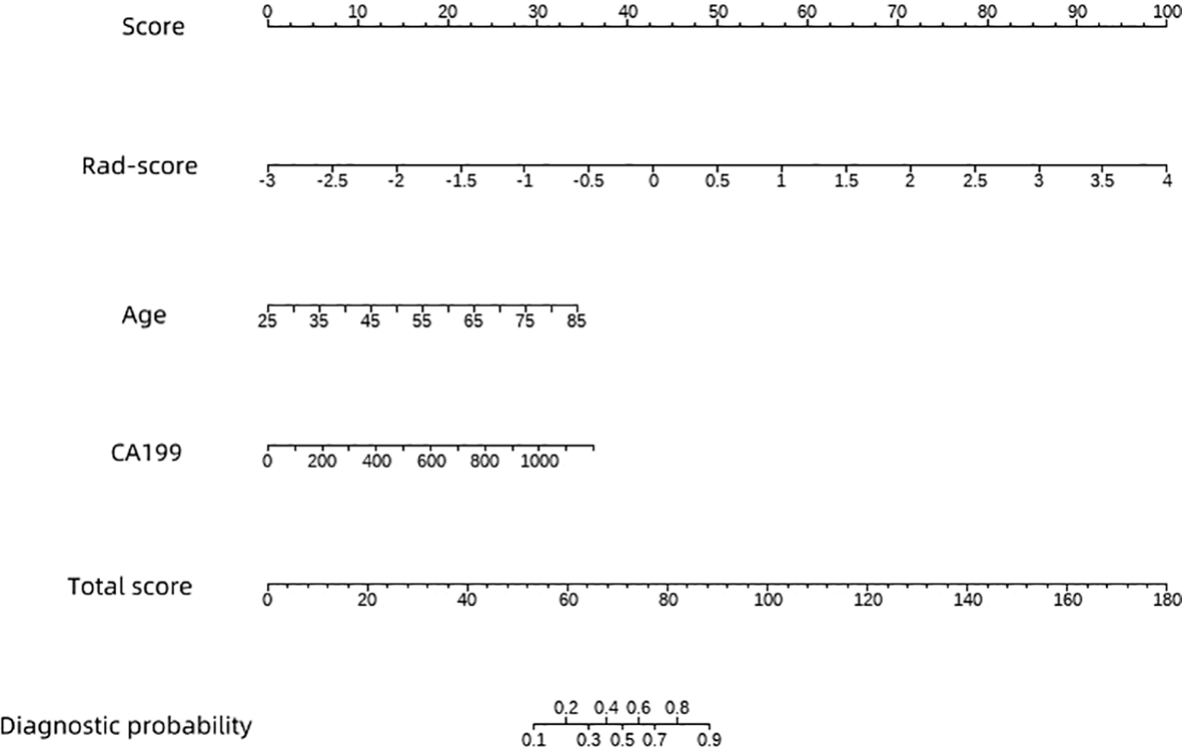
A nomogram model based on clinical features and rad-scores.

We conducted univariate logistic regression analysis using the rad-score of patients in the training set, combined with clinical data, laboratory test results, and EUS image features. Indicators with *p* < 0.1 (rad-score, age, CA199, lesion size, and cystic–solid appearance of lesions) were selected for multivariate logistic regression analysis. The results showed that the rad-score [OR = 5.254 (95% CI 2.409–14.85), *p* < 0.001], age [OR = 1.089 (95% CI 1.026–1.176), *p* = 0.012], and CA199 [OR = 1.003 (95% CI 1.001–1.007), *p* = 0.006] were significant factors in distinguishing between benign and malignant pancreatic lesions. These three indicators were used to construct a nomogram prediction model, as shown in **Figure 4**.

### 3.4 Validation and evaluation of model performance

**Figure 5:**
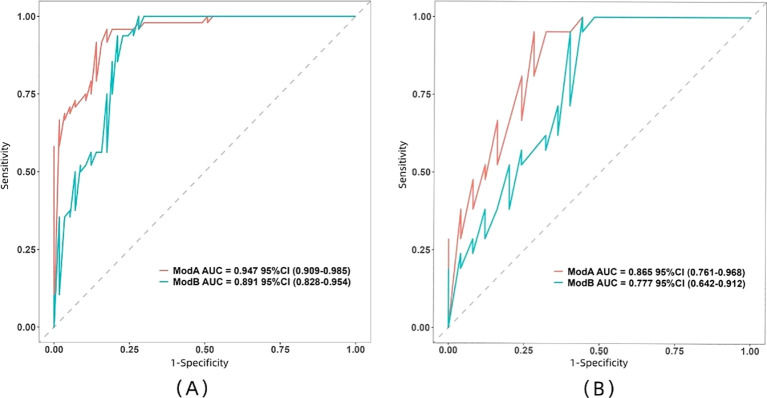
Comparison between the ROC curves of the two models in both training set **(A)** and validation set **(B)**. Model A, nomogram model; Model B, radiomics model.

**Figure 6:**
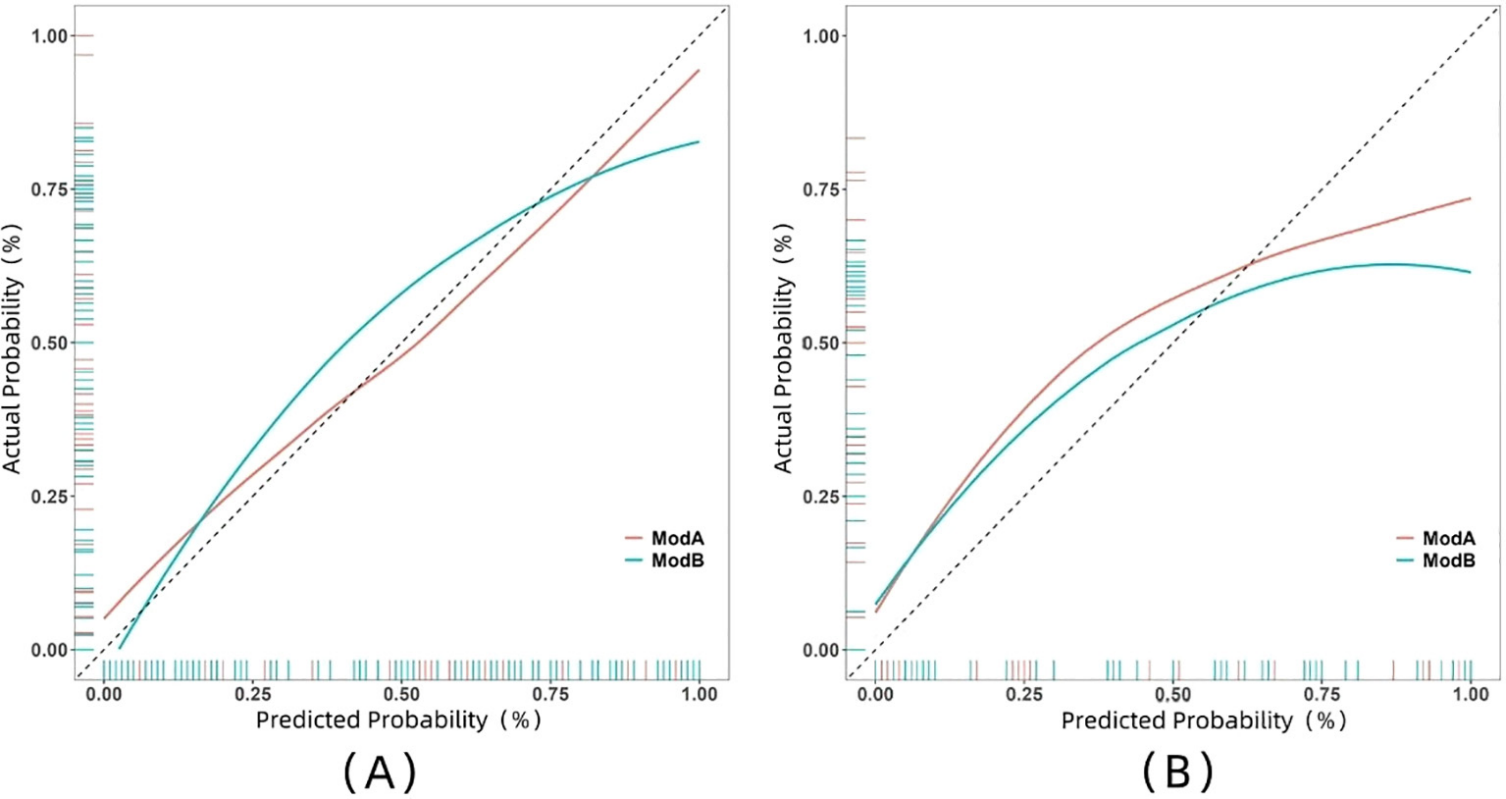
Calibration curves of two models in both training set **(A)** and validation set **(B)**. Model A, nomogram model; Model B, radiomics model.

**Figure 7:**
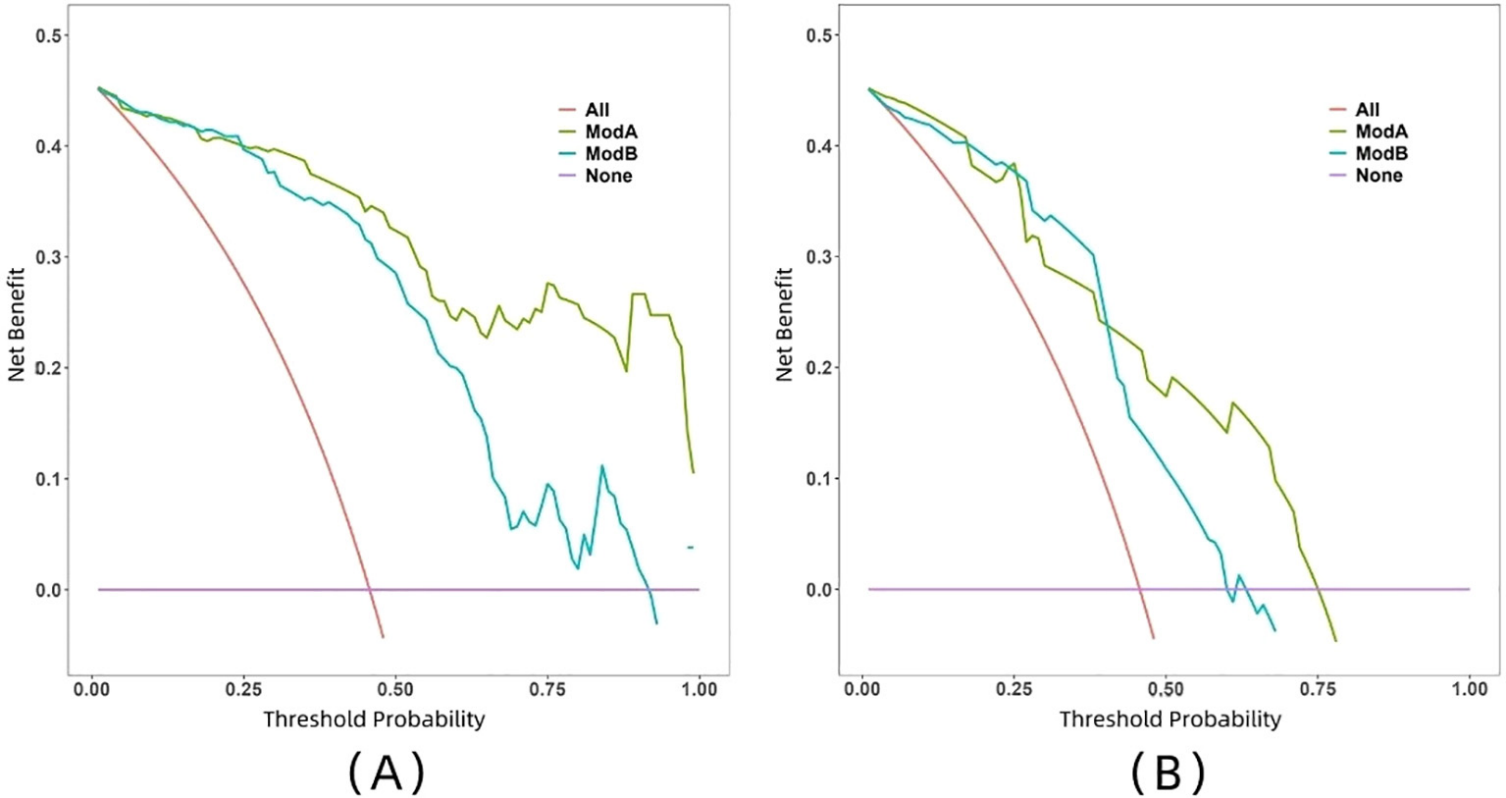
DCA curves of two models in both training set **(A)** and validation set **(B)**. Model A, nomogram model; Model B, radiomics model.

The ROC curve illustrates the relationship between sensitivity and specificity of the model at different thresholds and reflects the model’s ability to correctly distinguish between different patients. A higher AUC (closer to 1.0) indicates better discrimination. As shown in **Figure 5** and **Table 3**, the AUC of the nomogram prediction model in the validation set is higher than that of the single radiomics model (0.865 vs. 0.777), with a statistically significant difference (DeLong *p* < 0.05). The calibration curve evaluates the model’s consistency by comparing predicted risk with actual risk, where closer alignment to the ideal curve signifies better calibration. **Figure 6** demonstrates good consistency for both models in the training and validation sets. The DCA curve is used to evaluate the clinical net benefit of the model. As shown in **Figure 7**, when the threshold probability is between 0% and 75%, using the nomogram model to differentiate between benign and malignant pancreatic lesions in the validation set provides a higher clinical benefit than treating all lesions (assuming all lesions are malignant) or not treating all lesions (assuming all lesions are benign), and the net benefit is significantly higher than that of a single radiomics model within the threshold probability range of 50%–75%.

**Table 3:** Comparison of the diagnostic performance of the two models.

Model	Training set (*N* = 105)						Validation set (*N* = 46)					
	AUC (95% CI)	Accuracy	Sensitivity	Specificity	Positive predictive value	Negative predictive value	AUC (95% CI)	Accuracy	Sensitivity	Specificity	Positive predictive value	Negative predictive value
EUS radiomics model	0.891 (0.828–0.954)	0.857	0.938	0.790	0.790	0.938	0.777 (0.642–0.912)	0.67	0.762	0.600	0.615	0.750
Nomogram model	0.947 (0.909–0.985)	0.886	0.958	0.825	0.821	0.959	0.865 (0.761–0.968)	0.761	0.762	0.760	0.727	0.792

## 4 Discussion

The clinical manifestations of pancreatic lesions are similar; however, the treatment approaches and prognosis differ significantly between benign and malignant lesions. PC, characterized by its high malignancy and poor prognosis, requires early and timely diagnosis and treatment. Surgical resection is one viable treatment option. In contrast, benign pancreatic lesions generally have a better prognosis, with conservative treatment often being sufficient. Therefore, accurately distinguishing between benign and malignant pancreatic lesions is crucial for effective diagnosis, treatment, and prognosis. EUS is a pivotal examination method for diagnosing pancreatic lesions, which can perform comprehensive ultrasound scans of the entire pancreas with minimal interference. Its sensitivity in detecting pancreatic lesions is 93%–94%, higher than magnetic resonance imaging (MRI) (67%) and computed tomography (CT) (53%) (lesions < 30 mm) (22), and EUS can better detect lesions that have not been identified in CT or other imaging examinations (23). However, EUS is highly operator-dependent and presents technical challenges. According to the American Society for Gastrointestinal Endoscopy (ASGE), a comprehensive EUS practice should include at least 24 months of regular gastrointestinal endoscopy experience and at least 1 year of specialized training in biliopancreatic EUS (24). The high training costs and steep learning curve make it challenging for physicians to acquire expertise in EUS (25), and there is significant variability in diagnostic ability among different physicians, which limits the specificity and sensitivity of EUS diagnoses. Meanwhile EUS-FNA/FNB is an invasive examination and may increase the risk of complications. Therefore, it is extremely important to develop an accurate and non-invasive diagnostic method. To our knowledge, this study is the first to combine EUS radiomics with clinical information to construct the nomogram model for distinguishing benign and malignant lesions of the pancreas.

Our comparison of clinical data from patients with benign and malignant lesions revealed statistically significant differences in age and CA19-9 levels (*p* < 0.05). The age and CA199 levels of the patients in the malignant group were higher than those in the benign group. Multivariate logistic regression analysis also showed that age and CA199 were influencing factors in differentiating benign and malignant pancreas (*p* < 0.05). It has been shown (26, 27) that the incidence of PC increases with age, with almost 90% of cases diagnosed after the age of 55 years and the highest incidence above the age of 70 years. The results of this paper are consistent with it. In our study, the age of onset of the malignant group was approximately 63 years, which was significantly higher than that of the benign group, indicating that age is one of the factors affecting the development of PC. CA199 is the only FDA-approved clinical biomarker of PC, and it has suggestive significance in the diagnosis of PC, guiding treatment, and determining prognosis (28). Most patients with PC have significantly elevated serum CA199. The CA199 levels in malignant lesions of the pancreas were significantly higher than those in benign lesions in the training set of this paper, similar to previous studies, suggesting that CA199 levels are also helpful in identifying benign and malignant pancreatic lesions. However, the sensitivity and specificity of serum CA199 levels alone in differentiating benign and malignant pancreatic lesions are only 68% and 70%, respectively (29). Su et al. (30) also showed that elevated CA199 alone is not sufficient to differentiate PC from chronic pancreatitis, but when CA199 and other indicators are combined with radiomics information, the accuracy can reach over 80%, indicating that the combination of CA199 and radiomics can further improve diagnostic performance (31).

Radiomics is considered as an emerging non-invasive, objective, and efficient CAD technology by extracting high-throughput image features and transforming them into mineable data. These features can uncover biological information not visible through conventional imaging, improving disease diagnosis and prediction. Currently, some studies have demonstrated the promising results of CT-based and MRI-based radiomics in the identification of benign and malignant pancreatic tumors (32–35). However, compared to EUS, CT has a higher radiation dose and may cause allergic reactions to contrast agents, while MRI is time-consuming, expensive, artifactual, and not suitable for patients with ferromagnetic and/or metallic substances in the body (36). Therefore, there is growing interest in EUS-based radiomics analysis (7, 9, 37–40). In this study, we initially extracted 1,032 radiomic features from EUS images. Through ICC analysis, correlation analysis, and LASSO regression, we identified eight non-zero coefficient features, including one morphological feature, one first-order histogram feature, and six second-order histogram features (texture features). Morphological features describe the geometric characteristics of the image, reflecting the contour and size of the tumor, such as volume and maximum diameter. The first-order histogram feature describes the characteristics associated with the distribution of voxel intensities within the lesion image. Second-order histogram features (texture features) characterize the spatial distribution of voxel intensities, or the interrelationships between image gray values, reflecting the spatial heterogeneity within the tumor (41–43). Unlike traditional tumor imaging, which primarily relies on qualitative features such as tumor density, regularity of tumor margins, cellular composition within the tumor, and relationship with surrounding tissues, the radiomic features we extract can decode images into quantitative characteristics, including size, shape, and texture. This approach is more precise and reliable than qualitative diagnoses and can quantify image information that is undetectable by humans, aiding in the accurate identification of tumor benignity or malignancy (43, 44). Then, the rad-score of each patient was calculated using the above features, and the results showed that the rad-score of the malignant group was higher than that of the benign group in both the training and validation sets (*p* < 0.01). Multivariate analysis also showed rad-score as one of the influencing factors in identifying pancreatic benign and malignant (*p* < 0.05), similar to previous research findings (35). As the radiomics features can reflect the morphological characteristics of lesions and tumor heterogeneity, they can be used to identify the benign and malignant nature of pancreatic lesions. In this paper, the AUCs for our radiomics prediction model were 0.891 and 0.777 in the training and validation sets, respectively, with sensitivities of 0.938 and 0.762, and specificities of 0.79 and 0.6.

While radiomics models show good promise, they often operate in isolation, overlooking the potential influence of other factors such as laboratory tests and radiological date. The performance of the model needs to be improved. Consequently, many researchers have focused on developing multimodal models that combine radiomics with clinical information to improve diagnostic accuracy. Cui et al. (31) constructed a CNN model based on EUS images using information from 439 patients with solid pancreatic lesions, as well as a multimodal model that combined EUS images and clinical information. The results showed that the performance of the multimodal AI model was superior to that of the single model running solely on EUS images. Zhu et al. (45) demonstrated through multicenter external validation that the fusion model that integrated radiological and radiomics models outperformed models based on the clinical model and radiological model in predicting grade 1 and grade 2/3 non-functional pancreatic neuroendocrine tumors (NF-PNETs). Mo et al. (46) constructed an ultrasomics signature model, a clinical–ultrasonic signature model, and a combined nomogram model using ultrasomics signature and clinical–ultrasonic signature. The results showed AUC values of 0.649, 0.847, and 0.884 in the testing cohort, respectively, with the combined nomogram model showing the highest accuracy, effectively distinguishing between pancreatic neuroendocrine tumors and PC. However, the current studies lack visualization nomogram models that combine EUS radiomic features and clinical information to differentiate between a wide range of benign and malignant pancreatic lesions. Thus, we used EUS radiomics features and clinical indicators to construct a nomogram model, which was superior to a single radiomics model. The AUC and accuracy were 0.865 vs. 0.777 and 0.761 vs. 0.67, respectively, similar to the results of the above article. This suggests that the combination of the rad-score with relevant clinical indicators can provide a more comprehensive interpretation for the patient examination results and enhance the differentiation benign and malignant pancreatic lesions. Unlike the study by Mo et al. (46), our research, including various types of benign lesions with complex and diverse imaging, preliminarily classified pancreatic lesions into benign and malignant, not limited to the differentiation between pancreatic neuroendocrine tumors and PC. Additionally, when collecting clinical ultrasound features, we not only focused on characteristics such as age, lesion size, and ultrasound features, but also included the clinical indicator CA199 to construct the model, which yielded good results. In contrast to Cui et al. (31), who directly constructed models like CNNs, we developed a visual nomogram model to differentiate between benign and malignant pancreatic lesions. This model assigns corresponding scores to different values of each independent variable, allowing the prediction result to be obtained by calculating the total score. This approach is simple and convenient, making the results easier to understand and interpret. Moreover, it does not have algorithm patent barriers, allowing for broader application.

As pancreatic-occupying lesions are increasingly being detected, some controversial lesions inevitably require ultrasound-guided puncture biopsy for further diagnostic clarification. Although the diagnostic rate of the lesion is high (approximately 70%–82% for EUS-FNA and 70%–89% for EUS-FNB), it is an invasive operation. The incidence of post-puncture complications ranges from 1% to 2.5% (47, 48), and it is influenced by factors including lesion size, lesion location, type of puncture needle, and competence of the puncture (49). Our study showed that the nomogram model had an AUC of 0.825 and an accuracy of 0.761 in the validation set, which is similar to the diagnostic performance of puncture biopsy, and this method is non-invasive and not limited by puncture biopsy sampling. It may have important clinical significance by providing reliable supplementary information for EUS-FNA/B in the future.

This study is a preliminary attempt to identify benign and malignant pancreatic lesions based on EUS radiomics, which still suffers from the following shortcomings (1): This is a retrospective study with certain selection bias (2). This study is a single-center study with relatively small sample size, which poses challenges to the stability and reliability of the established model compared to models built on larger samples. The next step could involve expanding the sample size and increasing multicenter external validation to further enhance the robustness and generalizability of the model (3). In this study, two types of EUS devices and scanning methods were used to acquire images, and different scanning devices and scanning methods may have an impact on the study results. Meanwhile, the radial EUS scope used in this study was no longer widely used, which was also one of the limitations of this study (4). In this study, the ROIs were performed manually, which was complex and time-consuming, especially for lesions with unclear boundaries, and automated image segmentation algorithms can be developed or adopted in the next step to improve efficiency and reproducibility.

In summary, we established a radiomics prediction model and a nomogram model based on EUS images and clinical indicators to evaluate the diagnostic efficacy for benign and malignant pancreatic lesions. The results showed that the nomogram model was superior to the single radiomics prediction model in the differential diagnosis of benign and malignant pancreatic lesions and had better performance.

## Data Availability

The original contributions presented in the study are included in the article. Further inquiries can be directed to the corresponding author.
